# TMEM41B is a host factor required for the replication of diverse coronaviruses including SARS-CoV-2

**DOI:** 10.1371/journal.ppat.1009599

**Published:** 2021-05-27

**Authors:** Joseph D. Trimarco, Brook E. Heaton, Ryan R. Chaparian, Kaitlyn N. Burke, Raquel A. Binder, Gregory C. Gray, Clare M. Smith, Vineet D. Menachery, Nicholas S. Heaton

**Affiliations:** 1 Department of Molecular Genetics and Microbiology, Duke University School of Medicine, Durham, North Carolina, United States of America; 2 Division of Infectious Diseases, Duke University School of Medicine, Durham, North Carolina, United States of America; 3 Duke Global Health Institute, Duke University, Durham, North Carolina, United States of America; 4 Duke Human Vaccine Institute, Duke University School of Medicine, Durham, North Carolina, United States of America; 5 Department of Microbiology and Immunology, University of Texas Medical Branch, Galveston, Texas, United States of America; 6 Institute for Human Infection and Immunity, University of Texas Medical Branch, Galveston, Texas, United States of America; 7 Duke Cancer Institute, Duke University School of Medicine, Durham, North Carolina, United States of America; Icahn School of Medicine at Mount Sinai, UNITED STATES

## Abstract

Antiviral therapeutics are a front-line defense against virally induced diseases. Because viruses frequently mutate to escape direct inhibition of viral proteins, there is interest in targeting the host proteins that the virus must co-opt to complete its replication cycle. However, a detailed understanding of the interactions between the virus and the host cell is necessary in order to facilitate development of host-directed therapeutics. As a first step, we performed a genome-wide loss of function screen using the alphacoronavirus HCoV-229E to better define the interactions between coronaviruses and host factors. We report the identification and validation of an ER-resident host protein, TMEM41B, as an essential host factor for not only HCoV-229E but also genetically distinct coronaviruses including the pandemic betacoronavirus SARS-CoV-2. We show that the protein is required at an early, but post-receptor engagement, stage of the viral lifecycle. Further, mechanistic studies revealed that although the protein was not enriched at replication complexes, it likely contributes to viral replication complex formation via mobilization of cholesterol and other lipids to facilitate host membrane expansion and curvature. Continued study of TMEM41B and the development of approaches to prevent its function may lead to broad spectrum anti-coronavirus therapeutics.

## Author summary

The COVID-19 pandemic has highlighted the burden that emerging coronaviruses can place on global health systems. Due to difficulty in predicting the characteristics of future pandemic viruses, pan-coronaviral therapeutics are highly desired. While many antivirals directly target viral proteins to inhibit their functions, targeting broadly required host-dependency factors represents an orthogonal approach that may have the added benefit of lower susceptibly to the emergence of viral escape mutants. One limitation of this approach, however, is that essential host factors for viral replication must be identified and characterized prior to development of novel therapeutics. In this work, we performed a genome-wide host-dependency factor screen with the endemic seasonal human coronavirus HCoV-229E. We identified TMEM41B, an ER-resident protein, as a critical host-dependency factor for not only HCoV-229E but also distantly related coronaviruses as well. Mechanistic studies revealed that TMEM41B is likely required to facilitate appropriate membrane composition for the establishment of intracellular viral replication complexes. Thus, the conserved requirement for TMEM41B is likely due to the fact that all coronaviruses establish similar structures for RNA replication. Future studies understanding how to pharmacologically target TMEM41B and its associated lipid mobilization pathways may lead to the development of broad-spectrum anti-coronavirus therapeutics.

## Introduction

Human coronaviruses (HCoVs) can cause a spectrum of disease ranging from mild respiratory illness to lethal disease [1]. While the *Coronaviridae* family is diverse in terms of genomic configuration and host range, it has recently been divided into two subfamilies and five genera: the alphaletoviruses and the alpha-, beta-, gamma-, and delta-coronaviruses [2,3]. The CoVs that cause human disease fall into the alpha- and beta-coronavirus genera. While those associated with severe disease and pandemic outbreaks, such as SARS, MERS, and SARS-CoV-2 are beta-coronaviruses, HCoVs that typically cause a mild seasonal respiratory disease such as 229E, NL63, HKU1 and OC43 span both the alpha- and beta-coronavirus genera [4].

The current SARS-CoV-2 pandemic has highlighted the critical need for a more complete understanding of how HCoVs replicate and spread, and thereby induce disease. One way to identify potential therapeutic intervention points is to understand the human host factors that the virus must co-opt to complete its lifecycle [5]. Even though HCoVs encode relatively large RNA genomes, their coding capacity is dramatically less than their human hosts, and as such, the virus must repurpose host proteins to establish productive replication. It is also likely that even divergent HCoVs require some of the same key host factors as there are many conserved features of their lifecycle such as the reorganization and expansion of membranes to establish sites of replication [6,7]. Identifying these conserved factors and understanding their role in the viral replication cycle may lead to the development of broadly acting antivirals that could be used against current, and potentially even future, coronavirus outbreaks.

For this study, we performed a genetic screen with the seasonal alphacoronavirus HCoV-229E and identified the host factor TMEM41B as being critical for viral replication. Importantly, the genetically distant porcine deltacoronavirus (PDCoV) and the betacoronavirus SARS-CoV-2 displayed a similar TMEM41B dependence. We characterize the localization of TMEM41B during infection and provide evidence for a role promoting appropriate lipid localization in infected cells. Future work understanding how this host factor may be therapeutically targeted may lead to the development of broad-spectrum anti-coronavirus interventions.

## Results

**Fig 1:**
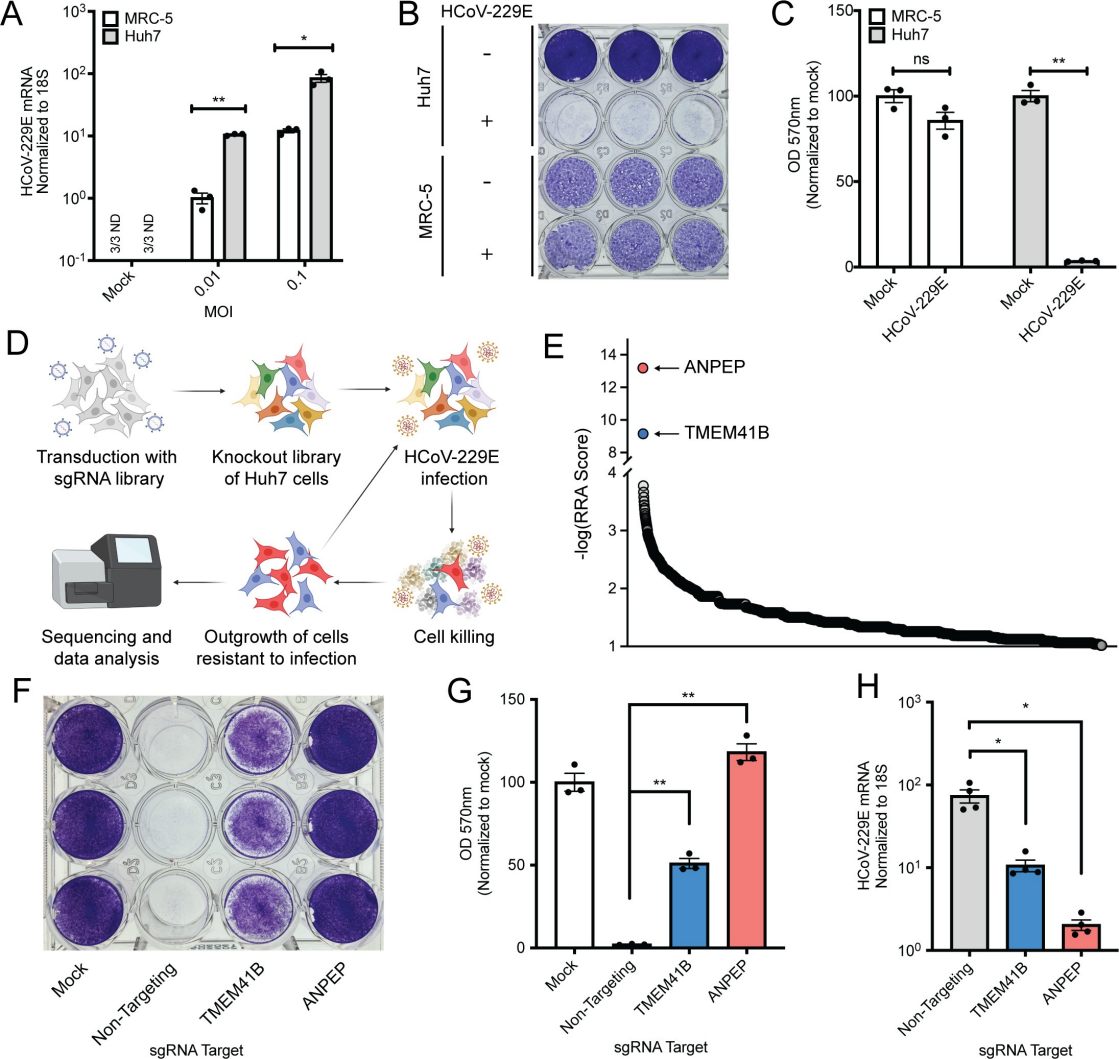
A genome-wide CRISPR/Cas9 screen with HCoV-229E identifies TMEM41B as a host-dependency factor. (A) MRC-5 and Huh7 cells were infected with HCoV-229E at the indicated MOI and viral RNA was quantified via qRT-PCR. N = 3. (B) Crystal violet staining of cell killing after 229E infection. MOI = 0.01, samples fixed and stained 72 HPI. (C) Quantification of B. Samples were solubilized in methanol and OD_570_ was read on a plate reader. N = 3. (D) Diagram of the screen design. (E) Representation of the top 2000 most enriched sgRNA targets after sequencing analysis. (F) Crystal violet staining of polyclonal cell populations transduced with sgRNAs targeting the indicated gene. MOI = 0.01, samples fixed and stained 72 HPI. (G) Quantification of crystal violet signal in polyclonal knockout cells after infection with HCoV-229E. MOI = 0.01, samples fixed and stained 72 HPI. N = 3. (H) qRT-PCR analysis of HCoV-229E RNA after infection of polyclonal knockout cells. MOI = 0.005, 24 HPI, N = 4. Error bars represent standard error measurement. Significance values were determined using a two-tailed, unpaired, Student’s t-test. *P<0.05, **P<0.001, ns = not significant, ND = not detected. For all panels except E, data are representative of at least two independently conducted experiments.

We selected the seasonal HCoV-229E, which contributes to the common cold disease burden [8], to screen for host factors that are required for HCoV replication. To facilitate the technical aspects of screening, we first wanted to identify a cell line highly susceptible to viral killing by measuring cytopathic effect (CPE). Based on previous reports of HCoV-229E *in vitro* culture systems [9,10], we tested MRC-5 (human lung fibroblast) and Huh7 (human hepatoma) cell lines for virally induced CPE. Although viral RNA was robustly detected in both cell lines, Huh7 cells harbored at least 10x more viral RNA and were much more efficiently killed by viral infection (**Fig 1A–1C**). We therefore moved forward with Huh7 cells as our screening cell line; Cas9 was stably introduced into the Huh7 cells and the GeCKO library of sgRNAs [11] were subsequently transduced at a low MOI such that the vast majority of cells received either zero or one sgRNAs. After antibiotic selection for those cells that had received an sgRNA, we infected the cells with HCoV-229E at an MOI of 0.02 and allowed multicycle amplification of the virus. Viral infection continued for more than two weeks until we isolated a cell population that was completely resistant to viral infection (**Fig 1D**). We reasoned that the long-term culture of virus with cells would lead to enrichment of a small number of host factors that most strongly suppressed viral infection or promoted host cell survival.

After Illumina sequencing and bioinformatic analysis [12] of the sgRNAs present in both the unselected and selected populations, we identified a number of sgRNAs significantly enriched in the post-selection pool. Two of the targeted genes, however, were enriched much more significantly than any of the others: aminopeptidase N (ANPEP), the known virus receptor for HCoV-229E [13,14] and an ER transmembrane protein TMEM41B, which has recently been reported to play roles in autophagy and lipid metabolism [15–17] (**Fig 1E and S1 Dataset**). Since TMEM41B was by far the highest magnitude predicted screen hit with an unknown function during HCoV-229E infection, we first attempted to validate it as a *bona fide* host factor. After cloning the TMEM41B sgRNA most enriched in the screen (as well as a non-targeting negative control and an ANPEP positive control sgRNA), we transduced the Cas9/sgRNA constructs into Huh7 cells. We then infected the resulting cell lines and, as predicted from our screening analysis, we observed protection from CPE when ANPEP and TMEM41B were targeted (**Fig 1F and 1G**) as well as a significant reduction in viral RNA levels (**Fig 1H**).

**Fig 2:**
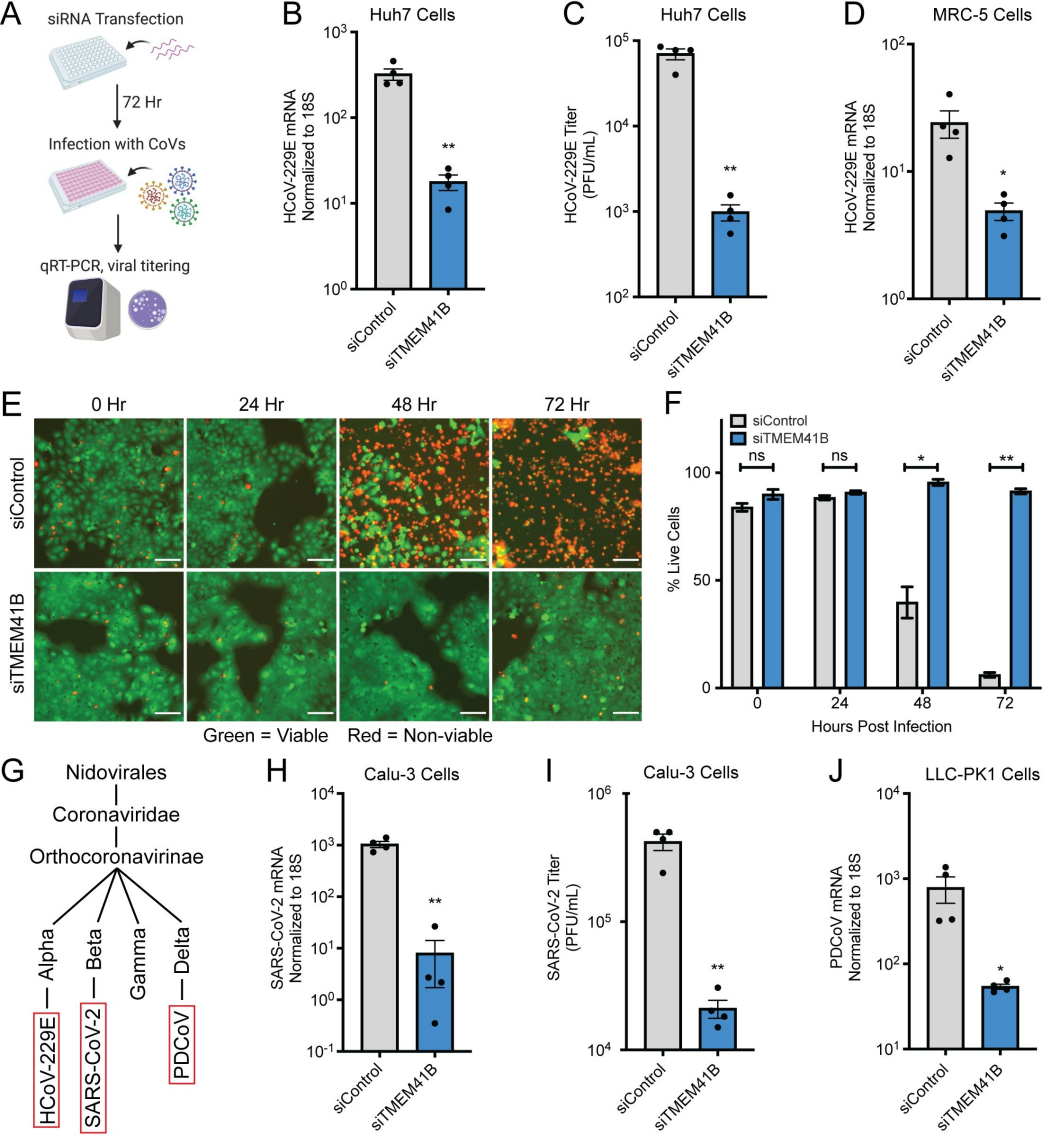
TMEM41B is essential for the replication of multiple coronaviruses. (A) Experimental scheme for siRNA-mediated targeting of TMEM41B. (B) HCoV-229E RNA quantified in siRNA-transfected Huh7 cells. N = 4. (C) HCoV-229E titer measured via plaque assay after infection of siRNA-transfected Huh7 cells. Supernatant was collected for titering 48 HPI, N = 4. (D) HCoV-229E RNA quantified in siRNA-transfected MRC-5 cells. (E) Live (green) and dead (red) cell staining after control or TMEM41B knockdown at the indicated timepoints. Scale bars are 100 μm. (F) Quantification of E. N = 3 independently captured images. (G) Simplified representation of the phylogenetic relationship between HCoV-229E, SARS-CoV-2, and PDCoV. (H) SARS-CoV-2 RNA quantified in siRNA-transfected Calu-3 cells. (I) SARS-CoV-2 titer measured via plaque assay after infection of Calu-3 cells. Supernatant was collected for titering 48 HPI, N = 4. (J) PDCoV RNA quantified in siRNA-transfected LLC-PK1 cells. Cells were infected with a dilution of PDCoV such that viral replication was detectable by qRT-PCR. Unless otherwise specified, MOI = 0.01 for all experiments and samples were collected at 24 HPI. Error bars represent standard error measurement. Significance values were determined using a two-tailed, unpaired, Student’s t-test. *P<0.05, **P<0.001, ns = not significant. Data are representative of at least two independently conducted experiments.

Next, as an orthogonal method to validate the requirement for TMEM41B, we utilized small interfering RNAs to reduce protein levels (**Fig 2A**). Knockdown of TMEM41B was efficient via this method (**S1 Fig**), and both HCoV-229E viral RNA (vRNA) and titer were significantly reduced compared to the non-targeting siRNA control in Huh7 cells (**Fig 2B and 2C**). We also verified viral sensitivity to the loss of TMEM41B in MRC-5 cells (a lung fibroblast line) to ensure the TMEM41B phenotype was not cell type specific (**Figs 2D and S1**). In order to understand when in the viral lifecycle TMEM41B may be required for viral infection, we evaluated cellular viability over an infection time course. We observed very little HCoV-229E mediated CPE at any timepoint, indicating that loss of TMEM41B was likely blocking some aspect of the initial infection and not spread of the virus (**Fig 2E and 2F**). Finally, we decided to test if the requirement for TMEM41B was shared across coronaviruses from different genera (**Fig 2G**). We therefore knocked down TMEM41B in human Calu-3 lung epithelial cells and porcine LLC-PK1 cells (**S1 Fig**) and infected with SARS-CoV-2 and PDCoV, respectively. For both viruses, which are genetically distinct from HCoV-229E, we observed a significant reduction in viral infection (**Fig 2H–2J**).

**Fig 3:**
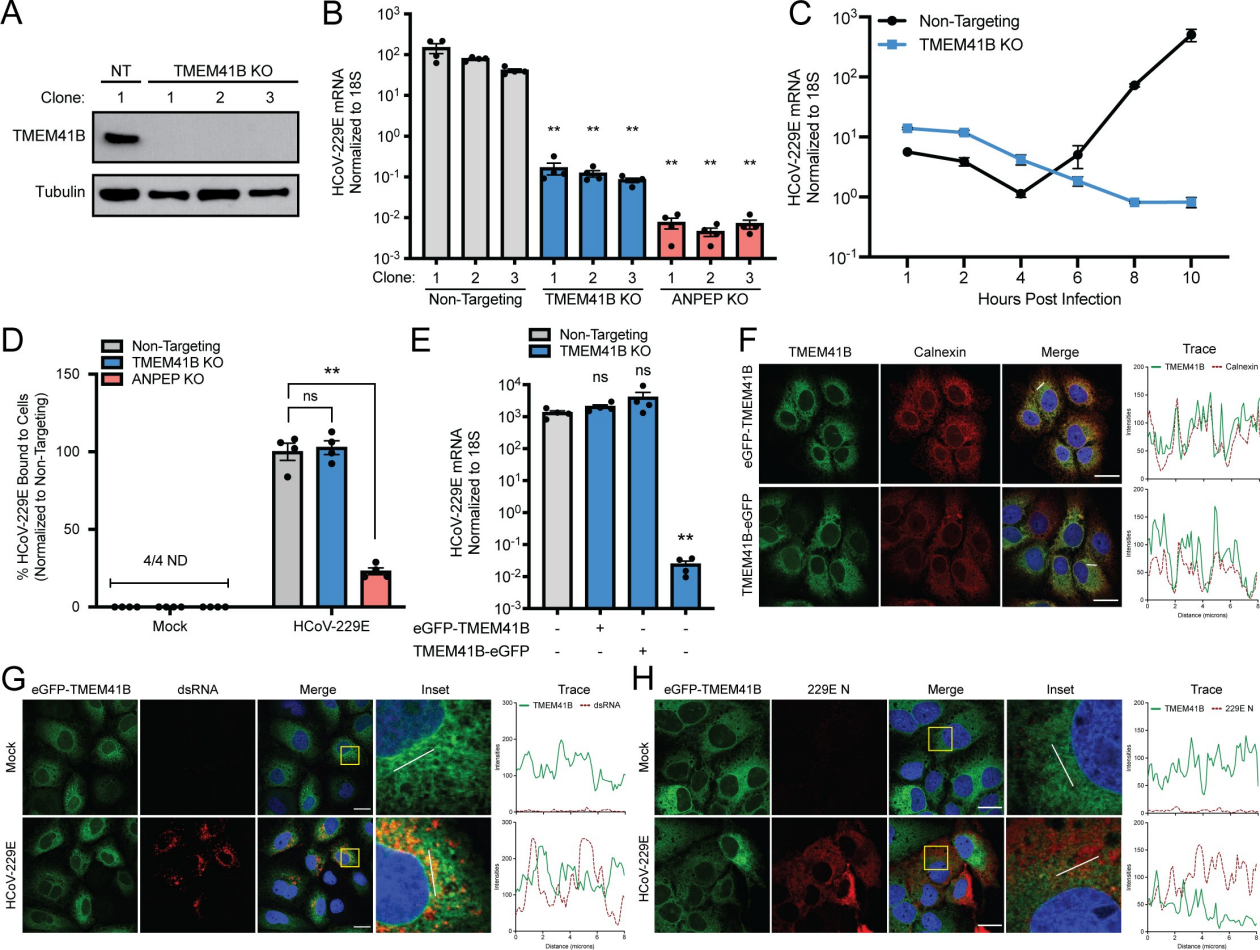
TMEM41B is required for the establishment of HCoV-229E replication. (A) Western blot for TMEM41B in the specified Huh7 clonal knockout lines. (B) HCoV-229E RNA quantified 24 HPI in knockout lines. Clone 1 from the non-targeting, TMEM41B, or ANPEP knockout line were used for the remainder of Fig 3. MOI = 0.01, 24 HPI, N = 4. Statistical significance was determined in comparison to non-targeting clone 3. (C) Non-targeting or TMEM41B KO cells were infected with HCoV-229E and RNA was quantified via qRT-PCR at the indicated timepoints. MOI = 1, N = 4. (D) HCoV-229E virus cold-binding assay in the indicated knockout cells. MOI = 1, N = 4. (E) HCoV-229E RNA quantified by qRT-PCR in TMEM41B knockout cells complemented with N- or C-Terminal eGFP-tagged TMEM41B delivered via lentivirus. MOI = 0.01, 24 HPI, N = 4. (F) N- or C-terminal eGFP-tagged TMEM41B colocalization with Calnexin in Huh7 cells. (G) N-terminal eGFP-tagged TMEM41B and dsRNA localization during HCoV-229E infection of Huh7 cells. MOI = 1, 24 HPI. (H) N-terminal eGFP-tagged TMEM41B and 229E N protein localization during HCoV-229E infection of Huh7 cells. MOI = 1, 24 HPI. All scale bars are 20 μm. White lines depicting linear trace regions are superimposed on corresponding merge and inset microscopy images for reference. Error bars represent standard error measurement. Significance values were determined using a two-tailed, unpaired, Student’s t-test. *P<0.05, **P<0.001, ns = not significant. Data are representative of at least two independently conducted experiments.

Next, we were interested in understanding how TMEM41B was broadly utilized by divergent coronaviruses. To facilitate mechanistic studies, we first generated clonal knockout cell lines. After sgRNA transduction and clonal purification, we isolated three independent lines each for: a non-targeting control sgRNA, a TMEM41B sgRNA, and an ANPEP sgRNA. Appropriate targeting was validated by sequencing of the edited loci which, in the cases of the TMEM41B and ANPEP clones revealed large in-frame or smaller frameshift-causing deletions (**S2 Fig**). Western blots for the TMEM41B clones revealed that, as expected, TMEM41B was reduced to below the limit of detection (**Fig 3A**). Infection of all nine cell lines with HCoV-229E revealed that, again, TMEM41B knockout significantly reduced viral RNA by 24 hours post-infection (by ~3 orders of magnitude) in a similar manner to loss of the viral receptor ANPEP (**Fig 3B**). With the cell lines validated, we returned to the question of when TMEM41B is required for viral replication. Since earlier experiments suggested the block was likely early, we performed a high-resolution time course of the early stages of HCoV-229E infection in TMEM41B knockout cells. Despite high or higher viral RNA levels present at 1 hour post-infection (HPI) (representing the initial viral inoculum) viral replication was undetectable during the first 10 hours of infection in TMEM41B KO cell lines (**Fig 3C**). This was in contrast to the non-targeted control cells, which showed active viral replication begin between 4 and 6 HPI (**Fig 3C**). In order to ensure the early viral RNA replication phenotype was not due to inhibition of virus binding to infected cells, we incubated virus with cells at 4°C for 1 hour (allowing binding but not viral entry), washed and then collected the cells followed by qRT-PCR for viral RNA. We failed to detect a difference between control and TMEM41B knockout cells while the ANPEP knockout cells showed a significant decrease in vRNA signal (**Fig 3D**). These data together indicated that loss of TMEM41B inhibited a stage in the HCoV-229E lifecycle post-receptor engagement, and prior to initiation of productive viral RNA replication.

The TMEM41B phenotype could potentially be explained by a failure to release viral RNA during the endocytic process or a failure to establish replication complexes in the ER. We attempted to distinguish between these two possibilities by visualizing the sub-cellular localization of TMEM41B. Commercially available antibodies against TMEM41B showed high levels of background staining in our knockout cell lines (**S3 Fig**). We therefore generated both N- and C-terminal fusions of eGFP to TMEM41B to monitor its localization. In order to ensure the fluorescent fusion proteins were functional (and presumably localized correctly), we showed that both of the constructs could effectively complement the viral inhibitory phenotype in a TMEM41B knockout cell line (**Fig 3E**). We next introduced both constructs into cells and observed a reticular localization pattern characteristic of the ER via confocal microscopy (**Fig 3F**), consistent with previous reports of TMEM41B localization [15–18]. Further, co-staining with the ER marker calnexin (particularly with the N-tagged construct) showed high, but not perfect co-localization, as TMEM41B has noticeably higher expression in the perinuclear region of the ER comparted to the periphery of the cell (**Fig 3F**).

It is known that coronaviral replication complexes are contiguous with the ER, and establishment of replication complexes is an essential and broadly conserved feature of replication for all members of the family [19]. Based on all of our previous data together, we hypothesized that the role of TMEM41B was likely in controlling some aspect of the formation of these replication structures as opposed to controlling some aspect of viral genomic release from the endosome. To better understand how TMEM41B was required for viral replication, we first wanted to define if it may be functioning as a structural component of the ER derived replication complexes. Infection of cells harboring our N-terminal TMEM41B construct however, revealed little overlap of TMEM41B with the vRNA replication marker double-stranded RNA (dsRNA). While the protein was not obviously excluded at sites positive for dsRNA (likely by virtue of these complexes being formed in the ER), there was no evidence for enrichment or relocalization of the protein (**Fig 3G**). Similar localization experiments with the viral nucleocapsid protein (which is present at, but not restricted to, replication complexes) also failed to reveal significant colocalization (**Fig 3H**).

In order to ensure that our inability to detect colocalization was not the result of the fluorescent tag location or the failure to detect relocalized endogenous TMEM41B, we repeated the dsRNA staining colocalization experiments with the C-terminal tagged construct and an antibody against endogenous TMEM41B. In both cases we again failed to detect appreciable enrichment of TMEM41B at sites positive for dsRNA (**S4 Fig**). While the relatively low levels of TMEM41B at replication complexes (or other virally induced cellular structures) could still be playing a structural role in either their assembly or maintenance, due to the magnitude of the TMEM41B knockout phenotype, we decided to look for additional ways that TMEM41B could be facilitating viral infection and replication.

**Fig 4:**
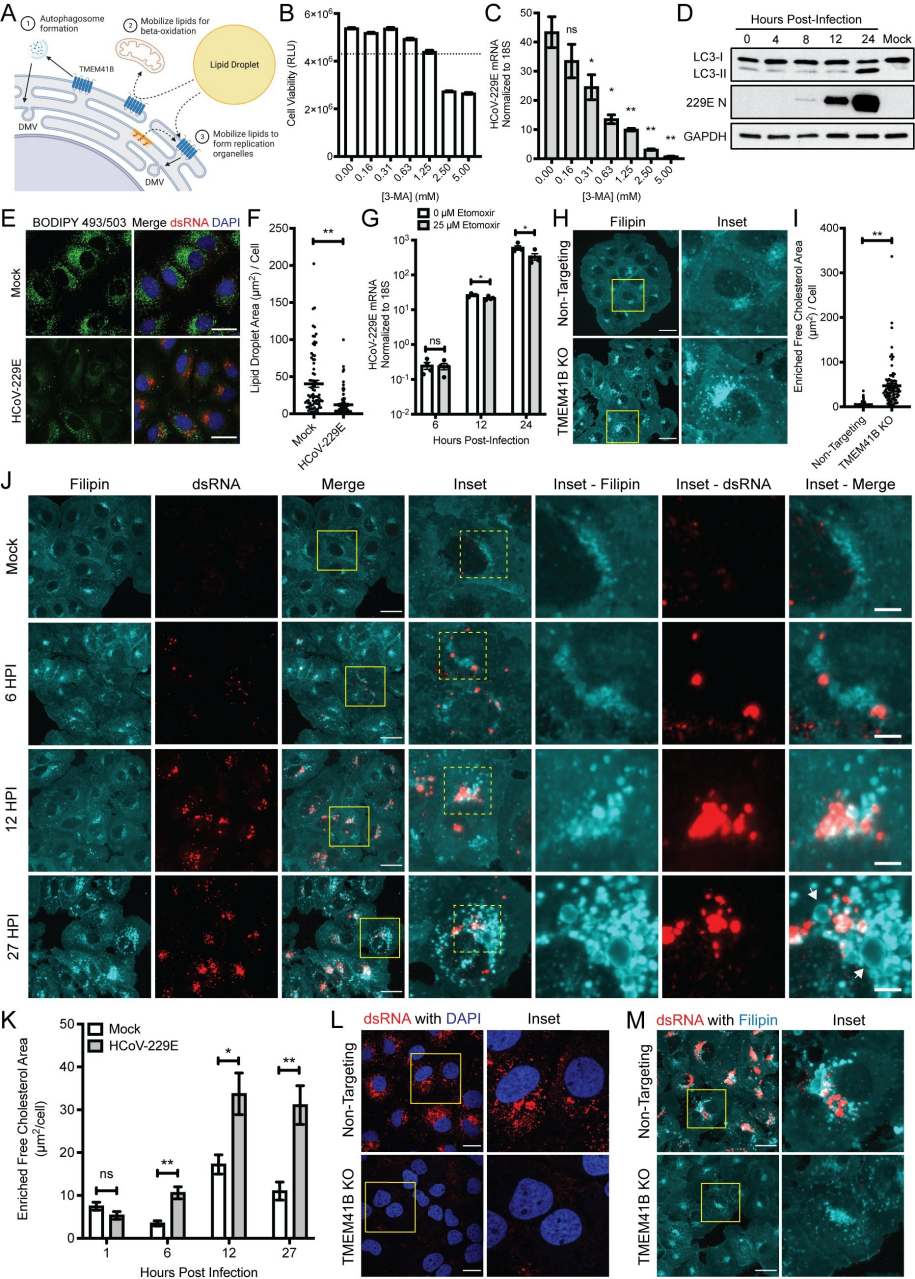
HCoV-229E remodels host cell lipid distribution in TMEM41B-associated functional pathways. (A) Schematic detailing possible roles for TMEM41B during coronavirus infection. (B) Huh7 cell viability determined via CellTiter-Glo (Promega) after treatment at indicated concentration of 3-MA for 24 hrs. N = 4. Dotted line marks 80% cell viability cutoff. (C) HCoV-229E RNA measured in Huh7 cells treated at indicated concentrations of 3-MA, added 1 HPI. MOI = 0.01, 24 HPI, N = 4. (D) Western blot for LC3 and HCoV-229E N protein during HCoV-229E infection. MOI = 1. (E) Lipid droplet size and localization in Huh7 cells infected with HCoV-229E. MOI = 1, 24 HPI. Scale bars are 30 μm. (F) Quantification of total lipid droplet area per cell from E. Mock, N = 77 cells; HCoV-229E infected, N = 85 cells. Values obtained from 5 independent images for each group. (G) HCoV-229E RNA measured at the indicated timepoints after infection with HCoV-229E. Etomoxir added 1 HPI at indicated concentration. MOI = 1, N = 4. (H) Filipin staining of non-targeting or TMEM41B KO Huh7 cells. Scale bars are 30 μm. (I) Quantification of accumulated free cholesterol area in cells from H. Non-targeting, N = 70 cells; TMEM41B KO, N = 82 cells. (J) Filipin staining of Huh7 cells during HCoV-229E infection at indicated timepoints. Arrows denote cholesterol-enriched vesicular structures observed at 27 HPI. MOI = 5. Scale bars in Merge panels are 30 μm, scale bars in Inset–Merge panels are 5 μm. (K) Quantification of enriched free cholesterol area during HCoV-229E infection in cells from J. Samples sizes for quantification were 1 HPI: mock, 50 cells, HCoV-229E, 68 cells, 6 HPI: mock, 74 cells, HCoV-229E, 55 cells, 12 HPI: mock, 75 cells, HCoV-229E, 87 cells, 27 HPI: mock, 59 cells, HCoV-229E 67 cells. (L) Staining of dsRNA in non-targeting or TMEM41B KO cells infected with HCoV-229E. MOI = 1, 24 HPI. Scale bars are 20 μm. (M) Filipin staining for free cholesterol in non-targeting or TMEM41B KO cells infected with HCoV-229E. MOI = 5, 27 HPI. Scale bars are 30 μm. All experiments with non-targeting or TMEM41B KO clones used clone 1 from the clonal lines generated in this study. Error bars represent standard error measurement. Significance values were determined using a two-tailed, unpaired, Student’s t-test. *P<0.05, **P<0.001, ns = not significant. Data are representative of at least two independently conducted experiments.

Although only recently characterized, TMEM41B has been reported to play several important roles in normal cell physiology (**Fig 4A**). Perhaps best characterized is the role of TMEM41B in facilitating the induction of functional autophagy [15–17]. However, it has also been reported to be important for the mobilization of lipids from cellular storage organelles known as lipid droplets [16,20]. Lipids released from lipid droplets (predominantly free fatty acids and cholesterol) are thought to be used in two main ways, either as fuel for cellular metabolism or incorporation into cellular membranes to facilitate membrane expansion and fluidity/bending. We therefore decided to see if we could detect TMEM41B-dependent roles during autophagy or for released lipids during coronaviral replication.

Viral induction of autophagy has been reported during coronavirus infection and studies have been published reporting both pro- and antiviral effects depending on the virus and experimental system [21]. Since a role for autophagy has not been fully resolved for HCoV-229E replication, we decided to first broadly inhibit autophagy with the well-characterized inhibitor 3-methyadenine (3-MA). Although administration of the drug affected cellular viability, we did observe a modest effect of the inhibitor on viral replication at 24 HPI (**Fig 4B and 4C**) suggesting the process was worth investigating further. Using lipidation of the protein LC3 as a marker for the induction of autophagy [22], we observed that indeed, autophagy appeared to be induced during HCoV-229E infection (**Fig 4D**). However, we failed to detect meaningful activation of the pathway prior to 24 HPI. Since we knew that the TMEM41B phenotype was detectible as early as 6 HPI, we rationalized that the loss of TMEM41B was likely not compromising viral infection primarily via suppressing the induction of mature autophagosomes.

We next turned our attention to potential roles of TMEM41B on mediating lipid mobilization from lipid droplets to facilitate viral replication. In agreement with previous studies [16,17,23], we observed that TMEM41B knockout significantly increased the size of lipid droplets in cells (**S5 Fig**), consistent with TMEM41B acting as a mediator of lipid mobilization. If significant lipid mobilization out of lipid droplets was indeed a critical process during viral replication, one would anticipate that the size of lipid droplets would likely decrease over the course of infection. Consistent with that idea, analysis for lipid droplets and viral infection markers revealed significantly decreased staining for storage lipids in infected cells (**Fig 4E and 4F**).

While mobilization of storage lipids could contribute to viral replication in a number of ways, it has been reported that the loss of TMEM41B compromised beta-oxidation, likely via the reduction in available pools of free fatty acids [16]. Beta-oxidation converts fatty acids into reducing equivalents such as NADH and FADH_2_ which can be used to generate ATP [24], which would presumably facilitate energy intensive viral replication. In order to test if beta-oxidation mediated energy production was important for HCoV-229E infection, we utilized the inhibitor etomoxir which prevents fatty acid import into the mitochondria. In a dose dependent manner that largely mirrored effects on cellular viability, we observed that HCoV-229E replication levels were suppressed by addition of the drug (**S6 Fig**). However, even relatively high concentrations of the drug (which likely have numerous off target effects [25,26]) did not inhibit HCoV-229E until at least 12 HPI, which again, is well past our observed phenotype for the loss of TMEM41B (**Fig 4G**). Similar to the autophagy experiments, while we cannot rule out that these processes (and potential TMEM41B dependencies) may not be important at some point during viral replication, we reasoned that lipid release to facilitate beta-oxidation was also not likely the primary role for TMEM41B during HCoV-229E infection.

Instead of contributing to the catabolism of lipids, TMEM41B could also potentially help traffic the free lipids and sterols released from lipid droplets (and/or other cellular sources) into appropriate cellular membranes and affect virus replication via that mechanism. Although there are limited specific detection reagents to track free lipids in cells, we chose filipin [27] to probe the localization of non-esterified cholesterol as a proxy for non-storage lipids during HCoV-229E infection. While filipin staining in control cells was fairly uniform, there was an apparent punctate and perinuclear accumulation of free cholesterol in TMEM41B knockout cells, suggesting loss of TMEM41B may broadly affect free lipid localization in addition to controlling trafficking from lipid droplets (**Fig 4H and 4I**).

In order to determine if TMEM41B-dependent alterations to cholesterol localization could potentially affect viral replication, we next tested if cholesterol localization/mobilization was altered during HCoV-229E infection in normal cells. Indeed, we observed that viral infection induced significant alterations to cholesterol localization as early as 6 HPI (**Fig 4J and 4K**). Later in infection, these cholesterol rich structures expanded to form large membrane enclosed vesicles reminiscent of virally induced replication complexes (**Fig 4J**). Staining for the viral dsRNA replication marker revealed that while filipin and dsRNA were enriched in the same areas of the infected cells; the association was sometimes proximal and sometimes directly overlapping, consistent with both viral replication and assembly complexes forming from areas of highly cholesterol enriched membranes (**Fig 4J**). Further, and as expected, we failed to detect any structures reminiscent of replication complexes (via use of both the dsRNA antibody and filipin staining) when TMEM41B was knocked out (**Fig 4L and 4M**). Together, these data suggest that replication complexes may not be able to form appropriately in TMEM41B knockout cells due to restricted access to the free cholesterol and other lipids required to expand and deform membranes. We therefore propose that a key role for TMEM41B during coronaviral infection is to facilitate an ER membrane lipid composition that is conducive to viral-mediated reorganization.

## Discussion

We designed a screening scheme in which competition between cells modified at different loci would enrich for a limited number of host factors with the strongest effects on HCoV-229E infection/replication. Outside of the HCoV-229E viral receptor, TMEM41B was our top hit which was not only important for HCoV-229E, but also PDCoV and SARS-CoV-2 across a variety of cell types. Confocal microscopy revealed that TMEM41B only partially localized with viral markers upon viral infection, and there was no compelling evidence for relocalization or accumulation of the protein at replication complexes. Looking for a non-structural role for TMEM41B during infection, we provide evidence that TMEM41B likely controls lipid trafficking which is important for virally-induced replication complex formation. Thus, future targeting of either TMEM41B directly or preventing lipid mobilization may serve as effective strategies to broadly inhibit coronavirus replication.

Ours is not the first report of the importance of TMEM41B during RNA virus infection. TMEM41B was identified as a hit in genome-wide host factor screens for the flaviviruses Zika virus (ZIKV) and dengue virus in 2016 and 2020, respectively [28,29]. It was also identified and validated as an important host factor for ZIKV after a viral/host proteomic interaction study in 2018 [30]. Despite being implicated for multiple flaviviruses, until very recently, there had been little follow-up or characterization. In January 2021, another study using genome-wide screening approaches for ZIKV and yellow fever virus host factors also identified TMEM41B as an essential host factor; in fact, it appears that TMEM41B is broadly required for most, if not all, flaviviruses [31]. Mechanistic studies with flaviviruses have revealed that TMEM41B physically interacts with the viral proteins NS4A and/or NS4B and is strongly relocalized to viral replication complexes [30,31]. Loss of TMEM41B leads to an exaggerated immune response in infected cells, which has been proposed to be due to compromised replication complex formation, potentially via inhibited lipid mobilization and the resulting inappropriate membrane composition at those sites [31].

Since the outbreak of SARS-CoV-2 and the spread of the COVID-19 pandemic, there has been considerable interest in defining host factors required for coronavirus replication. In parallel to this work, several other groups have independently identified TMEM41B as an important host factor for a number of coronaviruses including SARS-CoV-2, MERS-CoV, HCoV-OC43, HCoV-229E, and HCoV-NL63 [32–35]. Despite the repeated identification of TMEM41B, research into its contribution to viral infection has been limited to showing that TMEM41B has a post-entry phenotype and altered localization during infection with SARS-CoV-2. Our data agree well with these studies and also extend them to show that in addition to being required for alpha- and beta-coronaviruses, TMEM41B is also required for deltacoronaviruses. Further, using HCoV-229E as a model, we provide mechanistic evidence for how this protein may function during coronavirus replication, with at least one role being the mobilization of lipids during infection.

Despite this work, there remain many unanswered questions regarding the mechanism of the TMEM41B contribution to viral infection. These will be difficult to resolve, in no small part due to its loss imparting an early block in viral replication. As a result, essentially all virally induced processes are compromised regardless of whether or not they are TMEM41B dependent. Due to these limitations, our model that TMEM41B affects lipid composition at replication complexes is based on the convergence of multiple pieces of data. We show that global cellular lipid mobilization and cholesterol distribution is altered when TMEM41B is lost, and that cholesterol normally accumulates at sites of replication, consistent with reports of many other viruses that reorganize cellular membranes [36–38]. We believe it is likely that TMEM41B-mediated lipid mobilization is critical to physically expand host membranes and/or promote the membrane fluidity required for the extreme curvatures of the double-membrane vesicle-like replication complexes in the ER. However, because we cannot visualize any replication complexes when TMEM41B is lost, it is possible that a currently unknown, non-lipid mobilization role for TMEM41B prior to replication complex formation is in fact its critical function during viral infection.

Further, even within the context of altering lipid trafficking, changing the lipid content of membranes can, and likely does, affect multiple biological activities in the cell. Alterations to lipid levels, which has already been shown to occur after HCoV-229E infection [39], as well as the size and composition of lipid droplets can also influence diverse biological processes including innate immune responses [40–42] and their modulation could thereby indirectly affect viral replication. Additionally, the effects of TMEM41B on lipid mobilization may not be direct and other factors and pathways may also be required for its lipid mobilization function. We have also shown minor viral inhibitory effects from beta-oxidation and autophagy inhibitors, and while there is likely an early, primary role for TMEM41B during infection, there could be multiple, non-mutually exclusive TMEM41B-dependent cellular functions important for viral replication. Future work will be required to answer these questions, as well as define if different coronaviruses all require TMEM41B for the same biological activities during infection.

Interestingly, there appear to be key differences between the mechanism of flavivirus and coronavirus utilization of TMEM41B. In particular, flaviviruses induce strong relocalization of the protein via physical interaction with viral proteins [30,31]. At least for HCoV-229E, we observed limited colocalization with viral replication complex markers and multiple recent coronavirus co-immunoprecipitation proteomic studies have failed to detect high-confidence interactions between viral proteins and TMEM41B [43–45]. However, despite apparent differences in how the host factor is co-opted during infection, our lipid mobilization model for how TMEM41B contributes to coronavirus replication complex formation is ultimately similar to the model proposed for flaviviruses [31].

In sum, a more complete understanding of how coronaviruses hijack infected cells to facilitate viral replication will not only increase our understanding of viral pathogenesis but may also identify key intervention points to prevent viral infection and thereby disease. Our current study has highlighted one such factor that may be central to not only coronavirus replication, but to the replication cycle of other positive-stranded RNA viruses as well. Future work will be required to fully understand how TMEM41B contributes to the replication of different viruses, but this and subsequent translational work may help facilitate the development of truly broad-spectrum antivirals.

## Materials and methods

### Cell culture

MRC-5, Vero E6, Calu-3, and LLC-PK1 cell lines were obtained from ATCC. Huh7 cells were a kind gift from Dr. Emily Derbyshire. All cells were grown at 37°C with 5% CO_2_. Huh7 cells were maintained in DMEM with 10% FBS, and 1x Glutamax. MRC-5 and Vero E6 cells were maintained in MEM with 10% FBS, 1mM Pyruvate, and 1x MEM NEAA. Calu-3 cells were maintained in EMEM with 10% FBS. LLC-PK1 cells were maintained in M199 medium with 3% FBS. All cell media were supplemented with 1% Penicillin/Streptomycin and 2.5 mg/mL Plasmocin prophylactic (InvivoGen, ant-mpp) for the duration of the study. Huh7 cells were confirmed to be free of mycoplasma contamination.

### Virus stocks

HCoV-229E isolate VR-740 was obtained from ATCC and was grown in Huh7 cells in complete media (DMEM +10% FBS, Glutamax, 1% Pen/Strep) to generate the viral stock. Cells in 15 cm dishes were incubated with virus for 1 hr at 37°C, then total infection volume was brought up to 15 mL. Viral supernatant was harvested 48–72 hours post infection when CPE was observed then aliquoted and frozen at -80°C. Sequencing of the HCoV-229E nsp4 gene was used to verify the viral stock. Titer of the stock was determined via plaque assay on Huh7 cells. Briefly, cells were plated to near confluency in 6 well plates then inoculated with tenfold dilutions of virus in complete media. Cells were incubated with virus at 37°C for 1 hour, then cells were overlayed with DMEM, 10% FBS, 0.5% oxoid agar. Assays were incubated at 37°C for 72 hours, then fixed with 4% PFA, overlay was removed, and cells were stained with 0.1% crystal violet in dH_2_O. BEI isolate SARS-CoV-2 USA-WA1/2020 was grown on Vero E6 cells in viral growth media (MEM with 2% FBS, 1mM Sodium Pyruvate, 1x MEM NEAA, 1% Penicillin/Streptomycin). Infections were incubated for 1 hr at 37°C, then total infection volume was brought up to 30 mL. Viral supernatant was harvested ~72 hours post infection, then aliquoted and stored at -80°C. Consistent with previous reports of SARS-CoV-2 stocks amplified on Vero cells [46], Sanger sequencing revealed that our viral stocks partially harbored a small deletion in the furin cleavage site of the S protein. Titers of the viral stocks were determined via plaque assay on Vero E6 cells. A monolayer of Vero E6 cells in a 6 well plate was infected with tenfold dilutions of virus for 1 hour at 37°C. Virus was then removed and cells were overlayed with MEM with 2% FBS, 1mM Sodium Pyruvate, 1x MEM NEAA, 0.3% Sodium Bicarbonate, Glutamax, and 0.7% oxoid agar. Assays were incubated at 37°C for 72 hours, then stained with either 0.1% Crystal Violet in 10% Neutral Buffered Formalin or 0.05% Neutral Red. A PDCoV cell culture isolate (USA/IL/2014 strain, Lot#026PDV1402) was kindly shared via material transfer agreement with us at Duke as specimen “026-PDV” from the National Veterinary Service Laboratory in Ames, Iowa. LLC-PK1 cells in T75 flasks were inoculated with PDCoV for 1 hour, then media was replaced with serum-free M199 media supplemented with 0.2 μg/mL TPCK-treated trypsin (Sigma-Aldrich, T8802) and incubated at 37°C for 96 hours. Supernatant was then harvested, aliquoted, and frozen at -80°C.

### Virus infections

HCoV-229E infections were conducted in maintenance growth medium unless otherwise noted. SARS-CoV-2 infections were conducted in the appropriate cell medium with reduced serum (2% FBS). Viral inoculum was added to cells and cells were incubated with virus until the indicated timepoints unless otherwise noted. PDCoV infections were conducted in serum-free M199 medium supplemented with 0.2 μg/mL TPCK-treated trypsin.

### Plasmids

To tag TMEM41B with eGFP for fluorescence microscopy, TMEM41B was amplified from total Huh7 cellular RNA using SuperScript III RT-PCR System (Thermo, 12574026) then cloned into the pLEX plasmid via HiFi DNA assembly (NEB, E2621X). sgRNA sequences targeting TMEM41B (TATACTTACTCACTAAGCTG), ANPEP (CTACTGCAACGCTATCGCCC), or a non-targeting control (ATCGTTTCCGCTTAACGGCG) were constructed by annealing complementary oligos followed by ligation into lentiCRISPR v2 with T4 ligase (NEB, M0202L). lentiCRISPR v2 was a gift from Feng Zhang (Addgene plasmid #52961). For all generated plasmids, bacterial colonies were selected and plasmid DNA was isolated with the GeneJet Plasmid Miniprep kit (Thermo, K0503). Plasmid constructs were all confirmed using Sanger sequencing. To generate cell lines, lentiviruses pseudotyped with VSV-G were packaged using standard protocols and 1 mL of resulting lentiviral supernatant was used to transduce Huh7 cells. Huh7 cells were selected with 1 μg/mL puromycin after transduction.

### CRISPR screening, bioinformatic analysis, and sgRNA validation

Huh7 cells were transduced with lentivirus at high MOI to deliver lentiCas9-blast. lentiCas9-Blast was a kind gift from Feng Zhang (Addgene #52962). Cells were selected with 3 μg/mL blasticidin for several days. 3 x 10^7^ Huh7-Cas9 cells/replicate were transduced with CRISPR GeCKO Library A lentivirus at a MOI of 0.5 in 10 mL of complete media supplemented with DEAE dextran. 2 days post-transduction, cells were split into selection with 1 μg/mL puromycin and maintained and expanded in selection for 5 days to allow for gene editing and protein turnover. Half of the cells were collected for genomic DNA isolation to determine transduced input and the remaining ~1 x 10^8^ cells/replicate were infected with HCoV-229E at a MOI of 0.02. Cells were incubated for 48 hours to allow for complete killing, then all cells within each replicate were combined and replated. Dead cells were continuously washed away and cells were supplemented with complete media daily. After 13 days of outgrowth and infection, 2 x 10^7^ cells were collected for genomic DNA isolation. Genomic DNA was purified using QiaAmp Blood Maxi Kit (Qiagen, 51192). PCR was then performed on 18.5 μg of isolated genomic DNA using Ex Taq DNA polymerase (Takara, RR001). PCR gel bands were gel purified after running on a 3% agarose gel using the GeneJet gel extraction kit (Thermo, K0692). PCR products were quantified on an Agilent Bioanlyzer and samples were then sequenced on an Illumina MiSeq. Raw reads were extracted, read counts were normalized, and ranked gene scores and p values were determined and using the MAGeCK pipeline [12]. Analysis of normalized read counts in our transduced input indicated that 58609 and 58559 unique sgRNAs were detected in replicates 1 and 2, respectively, out of the total unique 65838 sgRNAs in GeCKO library A. Analysis of normalized read counts in our HCoV-229E-selected output indicated that 2250 and 3207 unique sgRNAs were detected in replicates 1 and 2, respectively.

### siRNAs and qRT-PCR

A Silencer Select siRNA targeting TMEM41B (s53991) was obtained from Thermo Fisher Scientific (Cat No. 4427037). A custom Silencer Select siRNA targeting porcine TMEM41B (s554786) was designed and obtained from Thermo Fisher Scientific (Sequence: GCAUAUUUCGCUACAUAUAtt; Cat No. 4399665). Silencer Select Negative Control No. 1 siRNA (Thermo, 4390843) was used throughout this study as a non-targeting negative control referred to as siControl. Huh7, MRC-5, Calu-3, or LLC-PK1 cells were transfected with siRNAs in 96 well or 24 well plates using HiPerfect transfection reagent (Qiagen, 301705) according to the provided protocol and incubated for 3 days at 37°C to allow for sufficient knockdown. To confirm knockdown, RNA was isolated using either NEB Monarch total RNA miniprep kit (NEB, T2010) or via TRIzol reagent resuspension followed by Zymo Direct-zol RNA miniprep kit (Zymo, R2072). One-step qRT-PCR was performed using the Invitrogen EXPRESS One-Step Superscript qRT-PCR kit (Thermo, 11781200) using commercial Taqman probes for TMEM41B (Thermo, Hs01379134_m1) and values were normalized to endogenous 18S rRNA using a 18S rRNA control mix (Thermo, 4319413E). All reactions were amplified on the Applied Biosystems QuantStudio 3 Real-Time PCR System and subsequently analyzed using QuantStudio software version v1.4.1.

### Viral quantification (RNA and titer)

After infection, total RNA was extracted from cells using the NEB Monarch total RNA miniprep kit or RNEasy 96 Kit (Qiagen, 74181) for HCoV-229E infections. For SARS-CoV-2 and PDCoV infections, cells were resuspended in TRIzol reagent followed by RNA isolation using either traditional phase separation with chloroform or Direct-zol RNA miniprep kit. HCoV-229E viral RNA was quantified using either a custom primer-probe set (FW: GGTAAGAGAGGTGGTGGTAATG, RV: TGGTCAACAAACTGCCATAAATC, Probe: /56-FAM/CATAACAGG/ZEN/TTTGCCATCGGCGC/3IABkFQ/) or a commercial Taqman probe (Thermo, Vi06439671_s1). SARS-CoV-2 viral RNA was quantified using a synthesized primer-probe set based on the sequences provided by the CDC “Research Use Only 2019-Novel Coronavirus (2019-nCoV) Real-time RT-PCR Primers and Probes” set N1. PDCoV viral RNA was quantified using a custom primer-probe set (FW: TCTACCCTCGTGCCACTATTA, RV: CTCTGTGATTTGCTTGCCTTTAG, Probe: /56-FAM/CAACTAAGC/ZEN/CTCTGTCTGCTGCCA/3IABkFQ/). All viral RNA was normalized to endogenous 18S rRNA. To determine HCoV-229E and SARS-CoV-2 titer, cells were infected for 1 hour at 37°C. Viral inoculum was then removed and medium was replaced with the appropriate fresh viral infection medium. Infections were incubated for 48 hours, then viral supernatant was collected and titer was determined via plaque assays.

### Cytopathic effect staining

To determine CPE, cells were infected with HCoV-229E at the indicated MOIs and timepoints. After infection, cells were washed once with 1x PBS, fixed with 4% PFA for 10 minutes at room temperature, washed 3x with 1x PBS, stained with 0.1% Crystal Violet in dH_2_O for 10 minutes at room temperature, then rinsed repeatedly with dH_2_O. Cells were then solubilized in methanol for 10 minutes, then quantified using a plate reader reading absorbance at OD 570nm. To perform live/dead fluorescent microscopy to evaluate CPE, cells were infected at the indicated MOIs and timepoints, stained with a live/dead cell imaging kit (Thermo, R37601), and imaged immediately on a ZOE fluorescent microscope.

### Western blotting

Cells were lysed via resuspension in RIPA buffer (10 mM Tris-HCl pH 7.5, 1 mM EDTA pH 8.0, 1% Triton X-100, 0.1% sodium deoxycholate, 140 mM NaCl, 0.1% SDS). Chromosomal DNA was sheared by passing lysates through an insulin syringe and cellular debris was removed via centrifugation (21,100g for 10 minutes at 4°C). Total protein concentration of cellular lysates was determined via Bradford assay and all samples were normalized to 1 μg/μL. Cellular lysates were separated by SDS-PAGE using 20 μg total cellular protein per lane (4–20% Mini-PROTEAN TGX gels (BioRad, 4561091), 120 V for 1 hour. Proteins were subsequently transferred to 0.45 μm nitrocellulose membranes (90 V for 1 hour at 4°C). Nitrocellulose membranes were blocked for at least 3 hours using PBST containing 5% milk. Membranes were incubated with primary antibody in PBST containing 5% milk overnight at 4°C. The following primary antibodies were used for protein detection: Sino Biological cat. no. 205880-T10 (TMEM41B; 1:1,000 dilution), Abcam cat. no. ab192890 (LC3B; 1:1,000 dilution), Cell Signaling Technology cat. no. 2118L (GAPDH; 1:10,000 dilution), and Sigma-Aldrich cat. no. T5168 (tubulin; 1:4,000 dilution). Anti-mouse (Invitrogen, A16072) and anti-rabbit (Thermo Scientific, A16104) secondary antibodies were used at 1:20,000 and 1:10,000 dilutions, respectively, in PBST containing 5% milk for 1 hour at room temperature. Blots were developed using Clarity or Clarity Max ECL substrates (BioRad, 1705060/1705062).

### Immunofluorescent microscopy staining and imaging

Glass cover slips were coated with poly-L lysine (Thermo, P4832), then rinsed once with sterile dH_2_O and allowed to air dry. Cells were plated on slides, then cultured or infected with HCoV-229E as described. Cells were then washed once with 1x PBS, fixed with 4% PFA at room temperature for 10 minutes, then washed 3x with 1x PBS. Samples were then permeabilized with 0.1% saponin for 20 minutes at room temperature, blocked in PBS + 5% BSA + 0.1% Saponin + 0.1% Tween-20 for 1 hour at room temperature, then incubated with primary antibody in antibody dilution buffer (PBS + 0.5% BSA + 0.1% Saponin + 0.1% Tween-20) overnight at 4°C. Samples were then washed 3x with PBS + 0.1% Saponin, then incubated with fluorescent secondary antibody for 1 hr at room temperature. Finally, samples were washed 3x with PBS + 0.1% Saponin and mounted on glass slides using Prolong Diamond Antifade with DAPI (Thermo). Primary antibodies used for microscopy include: Sino Biological cat. no. 205880-T10 (TMEM41B; 1:100 dilution), Sigma-Aldrich cat. no. HPA014946 (TMEM41B; 1:75 dilution), English and Scientific Consulting J2 anti-dsRNA monoclonal antibody (dsRNA, 1:200 dilution), Eurofins cat. no. M.30.HCo.I1E8 (HCoV-229E N, 1:100 dilution), Cell Signaling cat. no. 2433S (Calnexin, 1:50 dilution). Fluorescent secondary antibodies used include: Goat anti-Rabbit IgG (H+L) Cross-Adsorbed Secondary Antibody, Alexa Fluor 488 (Thermo, A-11008), Goat anti-Rabbit IgG (H+L) Highly Cross-Adsorbed Secondary Antibody, Alexa Fluor 647 (Thermo, A-21245), Goat anti-Mouse IgG (H+L) Cross-Adsorbed Secondary Antibody, Alexa Fluor 594 (Thermo, A-11005). All secondary antibodies were used at a 1:1000 dilution. All images were captured using a Zeiss 780 Upright Confocal microscope, except for samples stained with Filipin.

### Lipid droplet and cholesterol staining

Cells were plated on poly-lysine-coated glass coverslips. For staining of lipid droplets, fixed cells were stained with 2 μM BODIPY 493/503 (Thermo, D3922) in PBS at 37°C for 15 minutes, then immediately mounted with Prolong Diamond Antifade with DAPI and imaged with a Zeiss 780 Confocal microscope. When samples were co-stained with antibodies, cells were permeabilized with saponin and stained with antibodies followed by BODIPY 493/503 staining during secondary antibody incubation. For staining with NBD cholesterol, live cells were incubated with complete media supplemented with 2 μM NBD cholesterol (Thermo, N1148) for 15 minutes, then fixed, mounted and imaged. For Filipin staining of free cholesterol, fixed cells were stained with a solution of PBS + 0.05 mg/mL Filipin (Sigma-Aldrich, F9765) + 5% BSA at room temperature for 1–2 hours. For antibody staining with Filipin, cells were not permeabilized since Filipin permeabilizes cells. Primary and secondary antibody staining was completed after Filipin staining but in the absence of saponin. Samples were then mounted using Prolong Diamond Antifade (Thermo, P36965) and imaged on a Zeiss Axio Imager 2 fluorescent microscope.

### Image quantification

To determine fluorescent areas, fluorescence thresholds were set in Fiji and the analyze particles function was used. Unless otherwise noted, the sample size N represents individual cells collected from at least 3 independent images captured from the indicated sample. Live/dead staining was quantified using the detection of fluorescence maxima in Fiji.

### Drug treatments and cell viability

3-methyladenine (3-MA) (Sigma-Aldrich, M9281) was dissolved in complete media, sterile filtered, then frozen in aliquots at -80°C. Mock or infected cells were treated with the indicated concentrations of 3-MA at 1 HPI and incubated for 24 hours before determining cell viability with CellTiter-Glo (Promega, G7571) or quantifying viral RNA. (+)-Etomoxir sodium salt hydrate (Millipore Sigma, E1905) was dissolved in sterile dH_2_O and frozen in aliquots at -80°C. Mock or infected cells were treated with the indicated concentrations of Etomoxir at 1 HPI and incubated for 24 hours before determining cell viability via MTT assay or quantifying viral RNA. For the MTT assay, cells were incubated in serum-free medium supplemented with 2.5 mg/mL MTT reagent in PBS (Thermo, M6494) for 3 hours at 37°C, then lysed in MTT solvent (4mM HCl, 0.1% NP40 in isopropanol). OD 590 nm was read on a plate reader. All Etomoxir experiments were conducted in DMEM supplemented with 2% FBS.

### Statistical analysis

Significance values for all figures were determined using a two-tailed, unpaired, Student’s t-test. When RNA was not detected by qRT-PCR due to technical limitations, undetected replicates were omitted from statistical analyses. Statistical analysis was completed using GraphPad Prism 8. For all figures, error bars represent standard error measurement.

## Supporting information

S1 Dataset List of scored and ranked genes and associated p values from the CRISPR screen analysis. (XLSX)

S1 Fig siRNA-mediated TMEM41B knockdown in different cell types. (A-D) qRT-PCR quantification of TMEM41B mRNA in indicated cells transfected with a non-targeting control siRNA or a siRNA targeting TMEM41B. N = 4. Error bars represent standard error measurement. Significance values were determined using a two-tailed, unpaired, Student’s t-test. When RNA was not detected by qRT-PCR, undetected replicates were omitted from statistical analyses. *P<0.05, **P<0.001, ns = not significant, ND = not detected. Data are representative of at least two independently conducted experiments.
(TIF)

S2 Fig Sequence confirmation of clonal TMEM41B and ANPEP knockout lines. (A) Sequence confirmation of clonal TMEM41B knockout lines. (B) Sequence confirmation of clonal ANPEP knockout lines. Genomic DNA was amplified flanking the TMEM41B or ANPEP sgRNA target and cloned into a plasmid. The frequency of detected edit indicates how many plasmid clones out of the total sequenced for each knockout line harbored the detected editing pattern. No unedited wildtype sequences were detected for any of the TMEM41B or ANPEP KO clones used in this study.
(TIF)

S3 Fig Antibody detection of endogenous TMEM41B via immunofluorescent microscopy. Non-targeting or TMEM41B knockout cells stained with Anti-TMEM41B antibody obtained from (A) Sino Biological (Cat. 205880-T10) or (B) Sigma-Aldrich (Cat. HPA014946). All experiments with non-targeting or TMEM41B KO clones used clone 1 from the clonal lines generated in this study. Scale bars are 20 μm.
(TIF)

S4 Fig Detection of TMEM41B localization during HCoV-229E infection. (A) Localization of C-terminal tagged TMEM41B-eGFP and dsRNA during HCoV-229E infection. MOI = 1, 24 HPI. (B) Localization of antibody-detected endogenous TMEM41B (Sino Biological, 205880-T10) and dsRNA during HCoV-229E infection. MOI = 1, 24 HPI. White lines depicting linear trace regions are superimposed on corresponding inset microscopy images for reference. Scale bars are 20 μm. Data are representative of at least two independently conducted experiments.
(TIF)

S5 Fig Lipid droplet homeostasis is disrupted in TMEM41B KO cells. (A) Staining for lipid droplets (BODIPY 493/503) or esterified cholesterol (NBD cholesterol) in either non-targeting or TMEM41B KO cells. (B) Quantification of A. Sample sizes for BODIPY 493/503 stained samples: Non-targeting, N = 3749 lipid droplets, N = 2833 lipid droplets. NBD Cholesterol stained samples: Non-targeting, N = 1639 lipid droplets, TMEM41B KO, N = 4166 lipid droplets. Values obtained from 5 independent images for each group. All experiments with non-targeting or TMEM41B KO clones used clone 1 from the clonal lines generated in this study. Error bars represent standard error measurement. Significance values were determined using a two-tailed, unpaired, Student’s t-test. **P<0.001. Data are representative of at least two independently conducted experiments.
(TIF)

S6 Fig Etomoxir exhibits minor anti-viral activity against HCoV-229E. (A) Cell viability determined via MTT assay after 24 hours of treatment at the indicated concentration of Etomoxir. (B) Viral RNA quantified after treatment with the indicated concentration of Etomoxir at 1 HPI. 24 HPI, MOI = 0.01. Error bars represent standard error measurement. Significance values were determined using a two-tailed, unpaired, Student’s t-test. *P<0.05, **P<0.001, ns = not significant. Data are representative of at least two independently conducted experiments.
(TIF)
