# Supplementary figures and images for: TMEM41B is a host factor required for the replication of diverse coronaviruses including SARS-CoV-2
Source: PLoS Pathog. 2021 May 27;17(5):e1009599. doi: 10.1371/journal.ppat.1009599 (PMC8189496; doi:10.1371/journal.ppat.1009599)

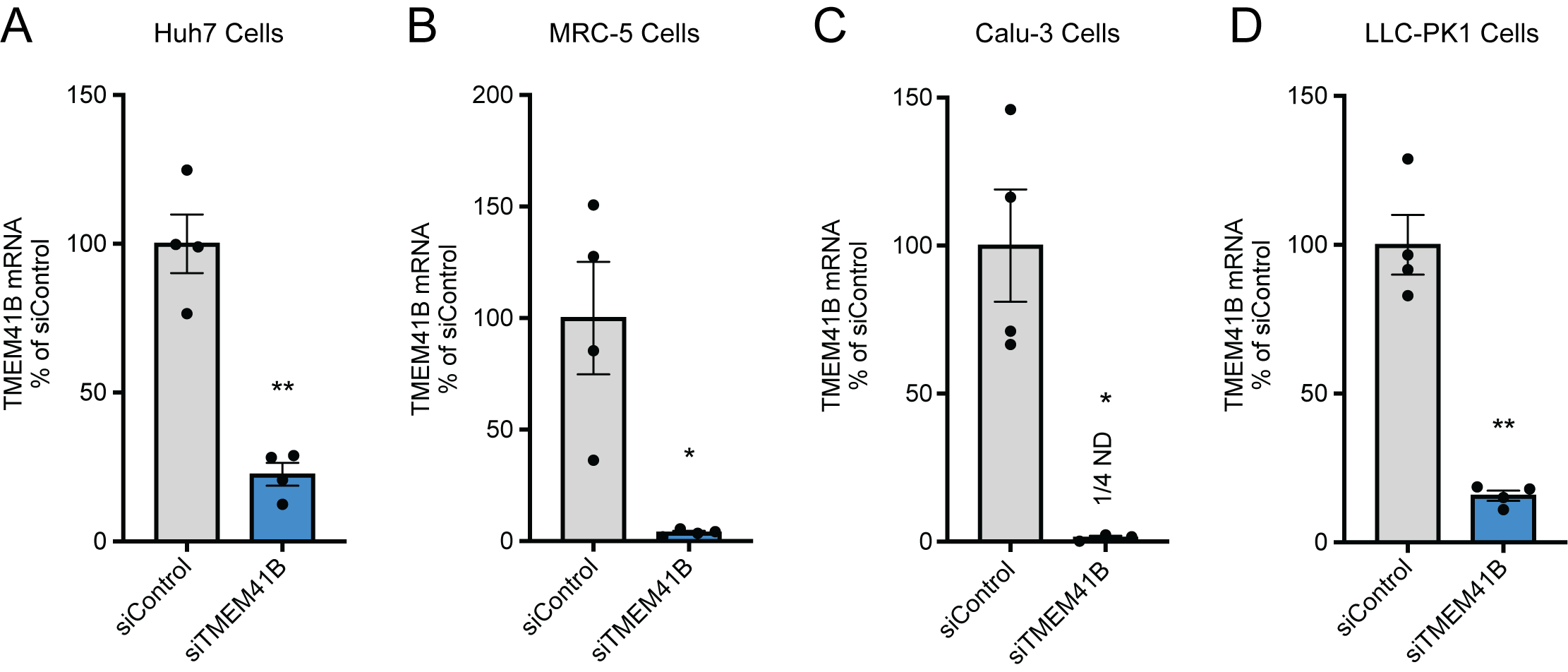

Supplement: S1 Fig — (A-D) qRT-PCR quantification of TMEM41B mRNA in indicated cells transfected with a non-targeting control siRNA or a siRNA targeting TMEM41B. N = 4. Error bars represent standard error measurement. Significance values were determined using a two-tailed, unpaired, Student’s t-test. When RNA was not detected by qRT-PCR, undetected replicates were omitted from statistical analyses. *P<0.05, **P<0.001, ns = not significant, ND = not detected. Data are representative of at least two independently conducted experiments. (TIF) [file ppat.1009599.s002.tif]

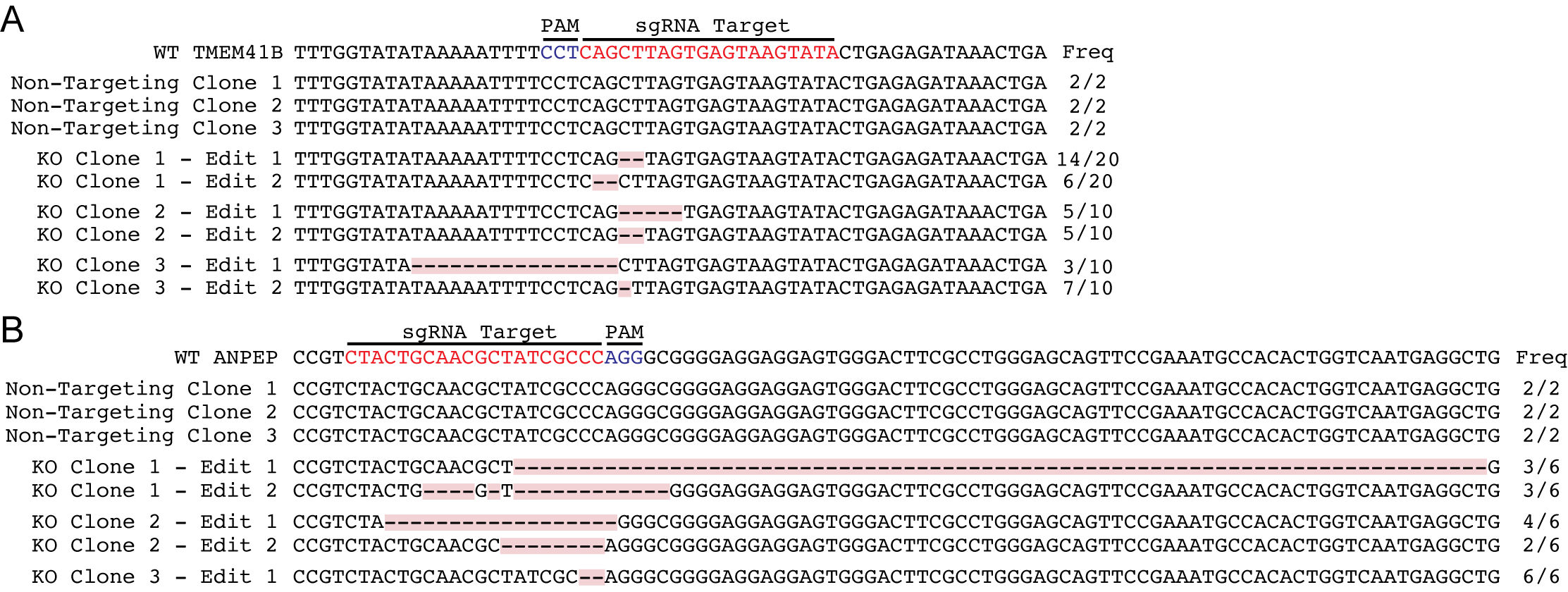

Supplement: S2 Fig — (A) Sequence confirmation of clonal TMEM41B knockout lines. (B) Sequence confirmation of clonal ANPEP knockout lines. Genomic DNA was amplified flanking the TMEM41B or ANPEP sgRNA target and cloned into a plasmid. The frequency of detected edit indicates how many plasmid clones out of the total sequenced for each knockout line harbored the detected editing pattern. No unedited wildtype sequences were detected for any of the TMEM41B or ANPEP KO clones used in this study. (TIF) [file ppat.1009599.s003.tif]

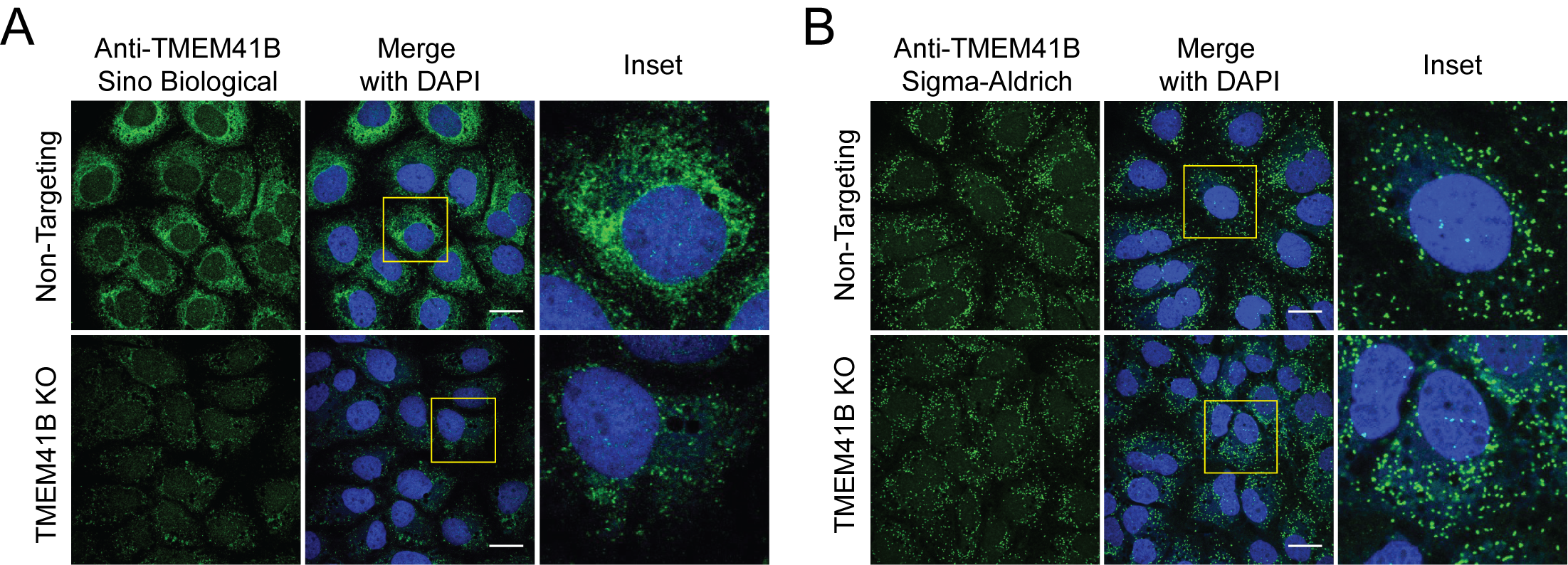

Supplement: S3 Fig — Non-targeting or TMEM41B knockout cells stained with Anti-TMEM41B antibody obtained from (A) Sino Biological (Cat. 205880-T10) or (B) Sigma-Aldrich (Cat. HPA014946). All experiments with non-targeting or TMEM41B KO clones used clone 1 from the clonal lines generated in this study. Scale bars are 20 μm. (TIF) [file ppat.1009599.s004.tif]

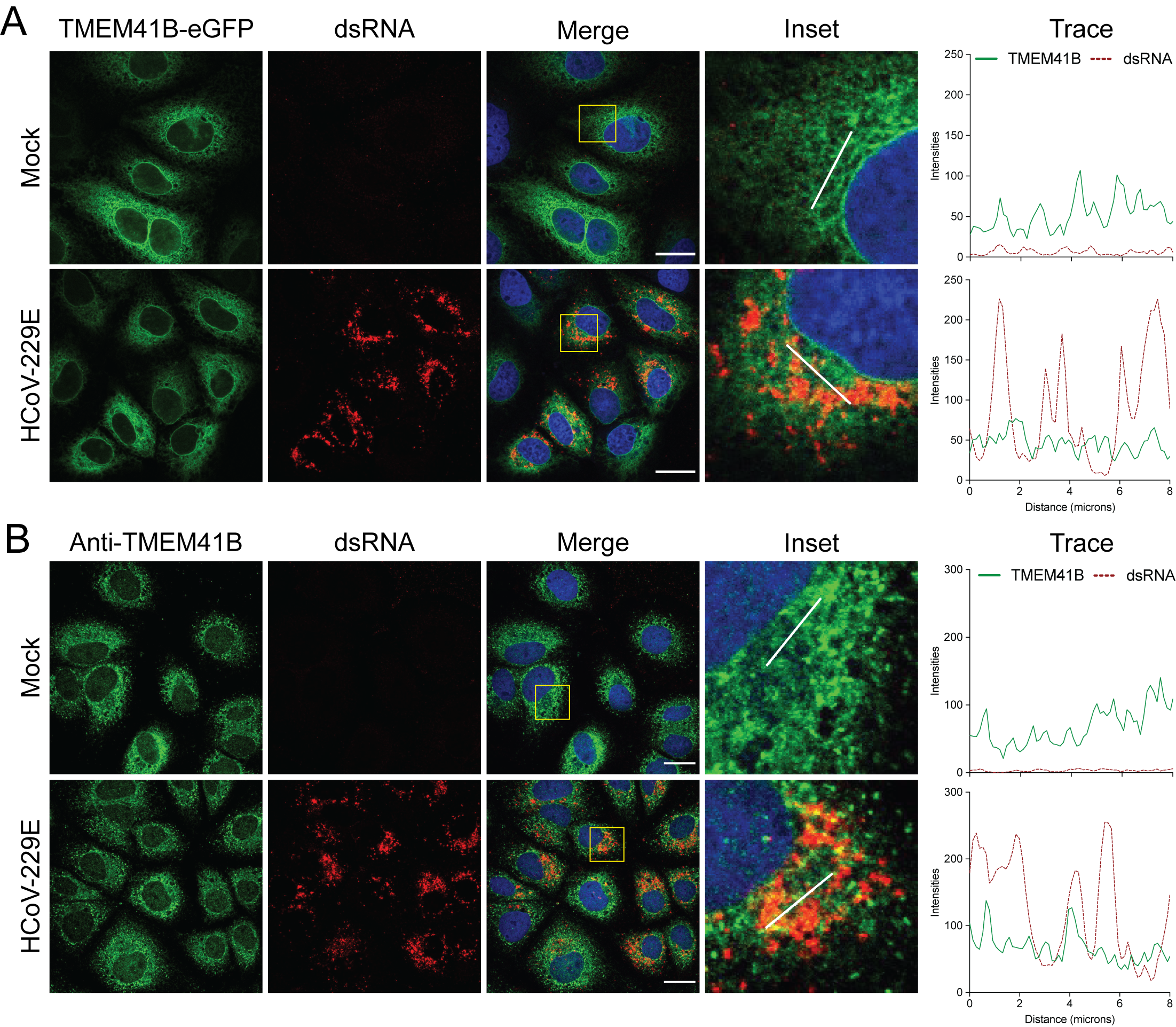

Supplement: S4 Fig — (A) Localization of C-terminal tagged TMEM41B-eGFP and dsRNA during HCoV-229E infection. MOI = 1, 24 HPI. (B) Localization of antibody-detected endogenous TMEM41B (Sino Biological, 205880-T10) and dsRNA during HCoV-229E infection. MOI = 1, 24 HPI. White lines depicting linear trace regions are superimposed on corresponding inset microscopy images for reference. Scale bars are 20 μm. Data are representative of at least two independently conducted experiments. (TIF) [file ppat.1009599.s005.tif]

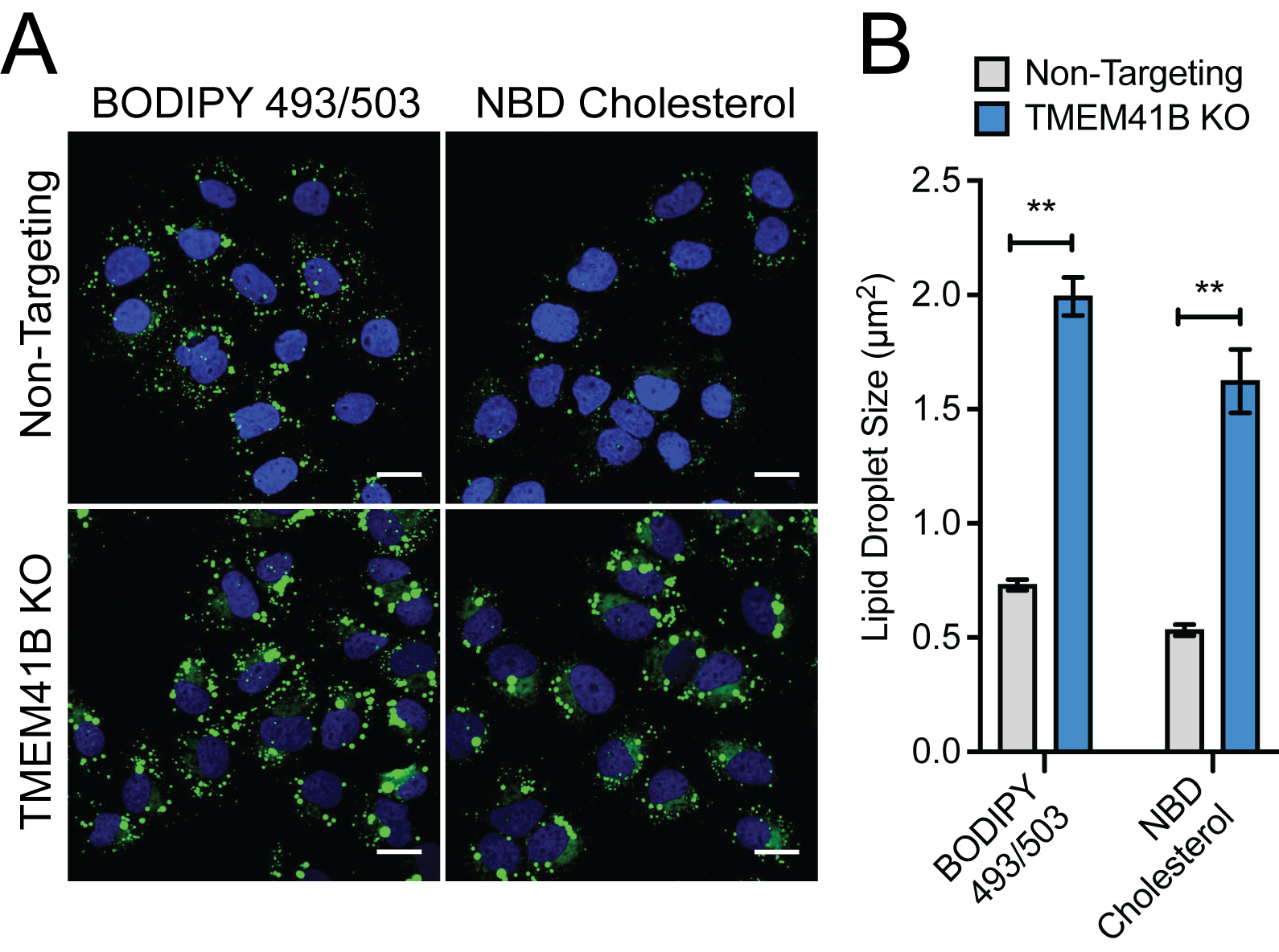

Supplement: S5 Fig — (A) Staining for lipid droplets (BODIPY 493/503) or esterified cholesterol (NBD cholesterol) in either non-targeting or TMEM41B KO cells. (B) Quantification of A. Sample sizes for BODIPY 493/503 stained samples: Non-targeting, N = 3749 lipid droplets, N = 2833 lipid droplets. NBD Cholesterol stained samples: Non-targeting, N = 1639 lipid droplets, TMEM41B KO, N = 4166 lipid droplets. Values obtained from 5 independent images for each group. All experiments with non-targeting or TMEM41B KO clones used clone 1 from the clonal lines generated in this study. Error bars represent standard error measurement. Significance values were determined using a two-tailed, unpaired, Student’s t-test. **P<0.001. Data are representative of at least two independently conducted experiments. (TIF) [file ppat.1009599.s006.tif]

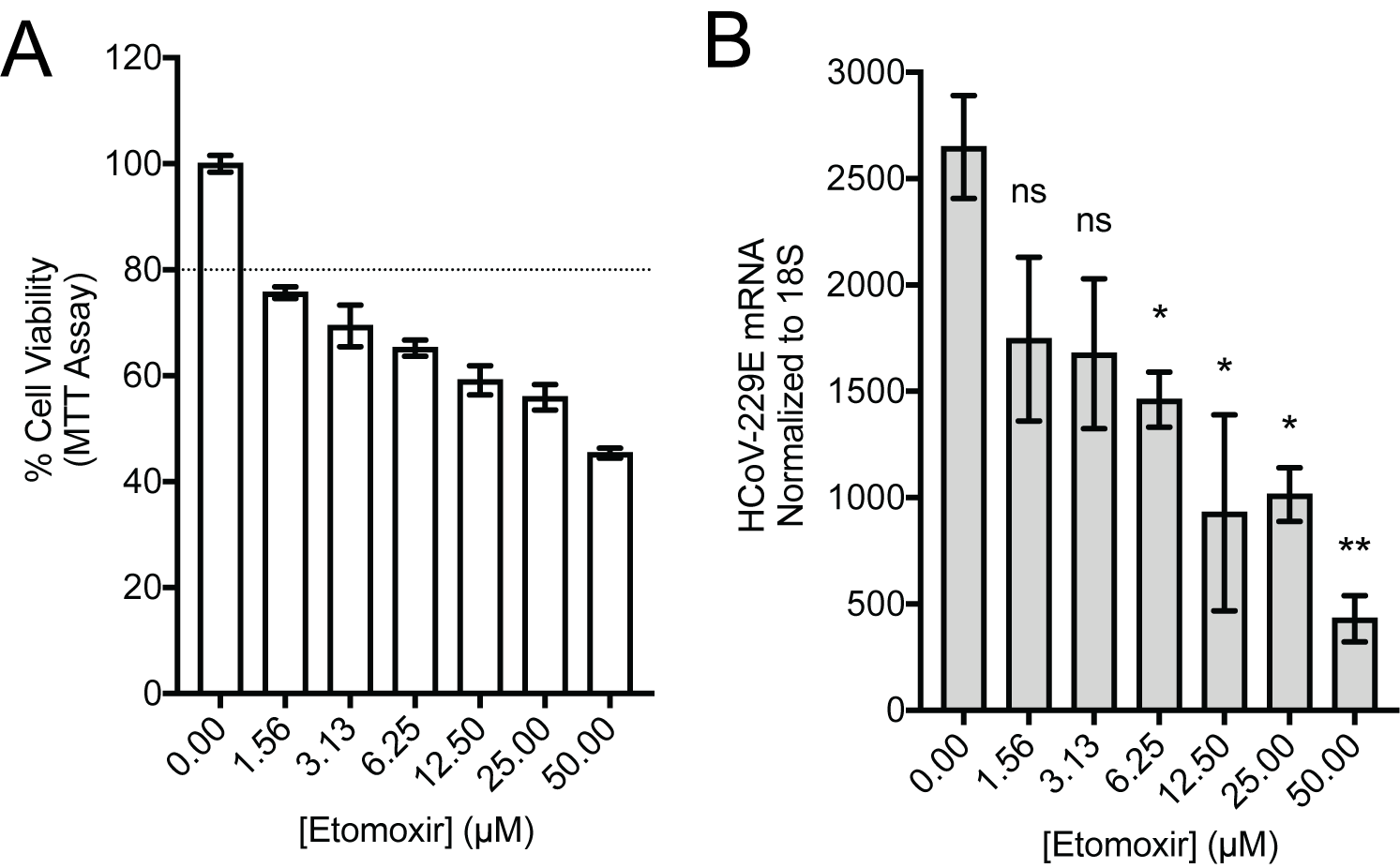

Supplement: S6 Fig — (A) Cell viability determined via MTT assay after 24 hours of treatment at the indicated concentration of Etomoxir. (B) Viral RNA quantified after treatment with the indicated concentration of Etomoxir at 1 HPI. 24 HPI, MOI = 0.01. Error bars represent standard error measurement. Significance values were determined using a two-tailed, unpaired, Student’s t-test. *P<0.05, **P<0.001, ns = not significant. Data are representative of at least two independently conducted experiments. (TIF) [file ppat.1009599.s007.tif]
